# Late vs. early intrauterine blood transfusion in fetal anemia: impact on maternal and neonatal outcomes

**DOI:** 10.3389/fmed.2025.1614989

**Published:** 2025-09-05

**Authors:** Adva Cahen Peretz, Lilah Tsaitlin-Mor, Gideon Leibner, Sarah M. Cohen, Danielle Amosi-Victor, Nitsan Haham, Tomer Shwartz, Nili Yanai, Shay Porat, Simcha Yagel, Dan V. Valsky

**Affiliations:** ^1^Hadassah Medical Center, Jerusalem, Israel; ^2^Faculty of Medicine, Hebrew University of Jerusalem, Jerusalem, Israel

**Keywords:** intrauterine blood transfusion (IUT), fetal anemia, hemolytic disease of the fetus and newborn (HDFN), late gestation IUT, neonatal morbidity, prematurity complications, alloimmunization

## Abstract

**Introduction:**

Optimal timing of final intrauterine transfusion (IUT) and delivery in fetal anemia remains controversial, balancing procedural risks against prematurity complications. Our objective is to evaluate the safety and effectiveness of extending IUT beyond 34 weeks gestation in appropriately selected cases.

**Methods:**

Retrospective cohort study comparing pregnancies receiving late IUT (≥34 weeks, *n* = 21) versus early IUT (<34 weeks, *n* = 31) at a single tertiary center (2005–2024). We analyzed 200 IUT procedures in 52 pregnancies. Late IUT was offered to stable cases without hydrops or previous significant complications. Primary outcomes included procedure-related complications and prematurity-related outcomes.

**Results:**

Late IUT showed no increase in procedure-related complications (0% vs. 20.0%, *p* = 0.069). Mean gestational age at delivery was higher in the late IUT group (37.2 ± 1.06 vs. 34.1 ± 3.6 weeks, *p* < 0.001), with reduced emergency cesarean rates (19% vs. 45%), higher birth weights (2,960 ± 399 g vs. 2,350 ± 620 g, *p* < 0.001), and lower NICU admission rates (29% vs. 71%, *p* < 0.05). These benefits persisted after adjusting for maternal characteristics. Subgroup analysis of hemolytic disease cases showed similar improvements with additional benefits in neonatal outcomes.

**Discussion:**

Extending IUT beyond 34 weeks in selected cases is safe and associated with improved obstetric and neonatal outcomes, supporting reconsideration of traditional gestational age limits for IUT.

## Introduction

Intrauterine intravascular transfusion (IUT) has revolutionized the management of fetal anemia, particularly in cases of severe red cell alloimmunization, parvovirus B19 infection, twin-to-twin transfusion syndrome, and other rare conditions (1–4). Despite its proven efficacy, IUT carries inherent risks including preterm labor, chorioamnionitis, emergency cesarean delivery, and rarely, fetal death. Reported complication rates range from 0.9 to 4.9%, (4–6) with fetal bradycardia being a significant concern that may necessitate immediate delivery (5). Post-IUT, neonates often require NICU admission for treatments like phototherapy, IVIG, or blood transfusions due to ongoing hemolysis and suppressed erythropoiesis (7, 8). Therefore, careful consideration is needed when deciding to perform IUT, determining the frequency of transfusions, and timing delivery.

The optimal timing of delivery in alloimmunized pregnancies remains controversial, particularly regarding the gestational age for the final IUT. Current American College of Obstetricians and Gynecologists (ACOG) guidelines suggest performing the last IUT between 30 and 32 weeks with delivery planned at 32–34 weeks following antenatal corticosteroids (9). However, growing evidence supporting the benefits of term delivery has led some experts to advocate for extending IUT up to 36 weeks when feasible (10, 11). The Society for Maternal-Fetal Medicine (SMFM) acknowledges this evolving practice, noting that while optimal delivery timing remains undefined, many clinicians now perform the final IUT at 34–35 weeks with delivery planned between 37 and 38 weeks (12).

This shift toward later delivery reflects two key developments: improved procedural safety in IUT and increasing recognition of the advantages of term delivery (13–17). Recent studies demonstrate that even modest extensions in gestational age at delivery can significantly improve neonatal outcomes, particularly regarding respiratory morbidity and neurodevelopmental outcomes (13, 14). However, this potential benefit must be weighed against the cumulative risk of repeated invasive procedures and the challenges of monitoring fetal anemia in late gestation (12, 15).

The OBGYN Fetal Therapy Units at Hadassah Medical Center have adopted an extended IUT protocol allowing procedures up to 36 + 6 weeks in selected cases, with deliveries typically scheduled between 37 and38 weeks. This approach aims to balance the benefits of term delivery against the risks of repeated procedures. By carefully selecting appropriate candidates and implementing enhanced monitoring protocols, we sought to evaluate whether extending IUT beyond traditional gestational age limits could safely reduce prematurity-related complications while maintaining procedural safety. Our findings could inform future clinical guidelines regarding optimal timing of both final transfusion and delivery in these complex cases.

## Materials and methods

### Study design

This retrospective cohort study employed a hierarchical testing strategy to evaluate intrauterine blood transfusions (IUT) performed at one of two fetal therapy units at Hadassah Medical Center from 2005 to 2024. The primary analysis assessed non-inferiority of late IUT (≥34 weeks) compared to early IUT (<34 weeks) regarding procedural safety, using a predetermined non-inferiority margin of 5%. Following establishment of non-inferiority, superiority analysis examined prematurity-related complications, including gestational age at delivery, preterm birth rate, NICU admission, low birth weight, and neonatal treatments for alloimmune anemia.

### Patient selection and study population

The study population comprised singleton pregnancies requiring IUT for fetal anemia, with indications based on middle cerebral artery peak systolic velocity exceeding 1.5 MoM and/or the presence of hydrops fetalis. We excluded cases of pregnancy termination, intrauterine fetal demise before 24 weeks, multiple gestations, and those with incomplete outcome data.

The decision to extend IUT beyond 34 weeks was made dynamically at approximately 34 weeks gestation through a standardized assessment process. Following the IUT performed around 34 weeks, we evaluated: fetal stability, i.e., absence of hydrops fetal is or signs of cardiac failure, biophysical score and Doppler measures within normal ranges; uncomplicated procedural course; and absence of maternal or obstetric complications requiring immediate delivery. Cases meeting all criteria were offered continuation of IUT beyond 34 weeks with planned delivery at 37–38 weeks.

### Procedure protocol

#### Operator standardization

All procedures were performed by four experienced operators (SY, NY, DVV, SP) using consistent protocols throughout the study period. Procedures were frequently performed collaboratively, ensuring standardization of technique and decision-making.

#### Technical protocol

All transfusions utilized O-negative, washed, irradiated, leukocyte-depleted blood, with CMV-negative status confirmed in most cases and serological cross-matching against donor samples. Repeat transfusions were indicated based on ongoing assessment of fetal well-being, particularly the persistence or recurrence of fetal anemia as determined by serial MCA-PSV measurements or the reappearance of hydrops signs. The frequency and timing of these repeat procedures were tailored to each case, considering factors such as the progression of anemia, gestational age, and overall fetal condition. Procedures were performed in fetal therapy units without regional anesthesia by one of four experienced operators. Pre-procedure blood sampling determined hemoglobin concentration and informed transfusion volume calculations. The use of prophylactic antibiotics and fetal paralytic agents (Atracurium, Vecuronium, or Pancuronium) was determined by clinical indicators and operator preference. Corticosteroids for fetal lung maturation were administered selectively based on anticipated preterm delivery risk.

#### Late IUT monitoring protocol

Late IUT cases underwent enhanced monitoring protocols adapted for late-gestation challenges. Given reduced MCA-PSV reliability after repeated transfusions, monitoring primarily utilized post-transfusion hematocrit levels with calculated fetal decline (average ~1% per day). Weekly surveillance included comprehensive ultrasound assessment, including biophysical score and Doppler measurements, cardiotocography, and maternal evaluation. Timing of subsequent transfusions was individualized to maximize intervals while preventing fetal anemia.

### Data collection and outcome assessment

Data collection integrated multiple sources including hospital electronic medical records (identified through ICD-9 codes), blood bank records, ultrasound unit documentation, and transfer records for deliveries at other centers. The primary safety outcome encompassed procedure-related complications during the last IUT in each pregnancy, defined as adverse events occurring within 1 week post-procedure (including PPROM, placental abruption, chorioamnionitis, or fetal demise) or within 24 h (including non-reassuring fetal monitoring, puncture site bleeding, or cord hematoma). Secondary outcomes focused on prematurity-related complications and neonatal interventions, with follow-up extending through 6 months postpartum. Neonates requiring IUT were managed by integrated maternal-fetal medicine and neonatology teams, with structured follow-up protocols extending through 6 months postpartum. All neonates remained within our health system, allowing complete capture of outcomes including late hemolytic anemia, hypogenerative anemia, and need for additional transfusions. Follow-up includes routine laboratory monitoring at our clinics post-delivery, with additional assessments as clinically indicated.

### Statistical analysis

The statistical approach began with non-inferiority testing using a 5% margin that was selected based on clinical risk–benefit considerations: given baseline IUT complication rates of 0.9–4.9%, a 5% absolute increase in procedural complications would be clinically acceptable to achieve the substantial benefits of term versus late preterm delivery demonstrated in our study population. Upon establishing non-inferiority, we proceeded with superiority testing for secondary outcomes. Continuous variables were analyzed using Student’s t-test or Mann–Whitney U test based on distribution normality, while categorical variables were assessed using Chi-square or Fisher’s exact test as appropriate. To account for potential confounding factors, we constructed multivariable logistic regression models adjusted for maternal age, parity, previous IUT occurrences, and gestational age at first IUT. All analyses were performed using R software (version 4.0.0).

This study was conducted in accordance with Declaration of Helsinki principles and received approval from our institutional review board (approval number HMO-0762-21).

## Results

### Study population and baseline characteristics

During the study period (2005–2024), 200 intrauterine blood transfusions were performed among approximately 230,500 deliveries at Hadassah Medical Center. After excluding cases with missing data (*n* = 4) due to delivery at outside hospitals without access to delivery and neonatal outcomes. The initial cohort included 62 pregnancies. We excluded five twin pregnancies: three cases of iatrogenic twin anemia-polycythemia syndrome following laser ablation for TTTS, two cases of single twin IUFD with surviving twin anemia, and one case of surviving twin anemia following selective reduction for IUGR. After excluding additional cases of pregnancy termination and early fetal demise, the final cohort comprised 52 pregnancies from 36 women, with 21 receiving late IUT (≥34 weeks) and 31 receiving their last IUT before 34 weeks.

**Figure 1:**
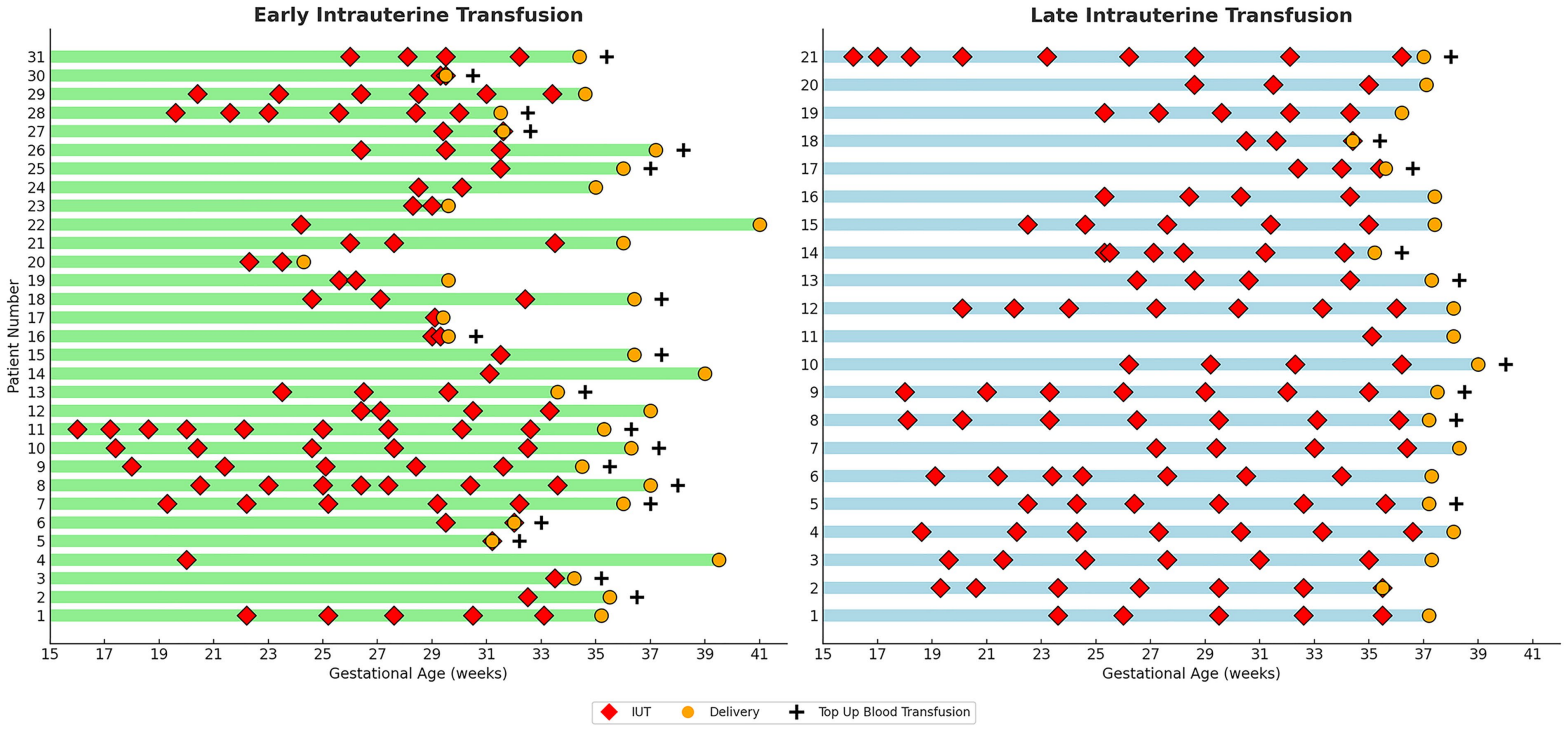
Characteristics of individual IUTs by study group. Graphic comparison of Individual IUTs by Study Group. Each row represents one pregnancy. Red diamonds (♦) indicate timing of individual IUT procedures; orange circles (●) indicate delivery timing; plus signs (+) denote cases requiring neonatal top-up blood transfusion. Green bars represent early IUT group (final IUT < 34 weeks); blue bars represent late IUT group (final IUT ≥ 34 weeks). Gestational age scale shown on x-axis (weeks).

In line with ACOG guidelines issued in 2012, our department adopted a trend toward full-term delivery around 2014, reflected in both delivery practices and fetal therapy protocols (Supplementary Figure 1). Throughout this transition, procedural techniques and operator performance remained consistent. The characteristics of each IUT in both groups are presented in Figure 1.

Baseline maternal characteristics were comparable between groups including maternal age, underlying conditions, parity, gravidity, and history of previous pregnancy complications (Table 1). The primary indication was Rh-D antibody (71% late IUT vs. 55% early IUT), followed by Kell antigen (19% vs. 10%), with parvovirus and chorioangioma cases occurring exclusively in the early IUT group. While gestational age at diagnosis and first IUT were similar between groups, the late IUT group had significantly higher total IUT numbers and later final procedures (Table 2).

**Table 1:** Baseline characteristics and history of pregnancy complications in late vs. early intrauterine transfusion groups.

Study group	Last IUT ≥34 weeks of gestation; *n* = 21	Last IUT<34 weeks of gestation; *n* = 31	*p*-value
Maternal age	29.3 ± 3.5	30.9 ± 6.2	0.4
Maternal background chronic diseases*	9.5% (2)	9.7% (3)	>0.9
Gravidity	5.4 ± 2.1	4.9 ± 2.7	0.5
Parity	3.5 ± 1.9	2.9 ± 1.7	0.4
History of IUT in previous pregnancy	48% (10)	26% (8)	0.1
History of neonatal transfusion	52% (11)	39% (12)	0.4
History of hydrops in previous pregnancy	33% (7)	13% (4)	0.1
History of IUFD	14% (3)	13% (4)	>0.9

Data are presented as % (n) or mean ± SD; Significance for differences was measured using the chi-square test, with Fisher’s exact test applied when expected frequencies were less than 5. *Maternal background chronic diseases include hypothyroidism, epilepsy, spherocytosis. IUFD, Intra-uterine fetal demise; IUT, intra uterine transfusion.

**Table 2:** Timing and indications for intrauterine transfusions: late vs. early IUT groups.

Study group	Last IUT ≥34 weeks of gestation; *n* = 21	Last IUT<34 weeks of gestation; *n* = 31	*p* value
IUT indication			
RH Isoimmunization	71% (15)	55% (16)	0.3
Kell Isoimmunization	19% (4)	10% (3)	
Parvovirus	0% (0)	10% (3)	
Chorioangioma	0% (0)	10% (3)	
Other indications*	9.5% (2)	19.3% (6)	
Maternal exposure to blood transfusion	24% (5)	6.5% (2)	0.1
GA at diagnosis of fetal anemia	23.0 ± 4.7 (15.6–34.0)	25.1 ± 5.1 (16.0–33.3)	0.2
GA at first IUT	23.8 ± 5.2 (16.1–35.1)	25.6 ± 4.9 (16.0–33.5)	0.2
Hydrops during pregnancy	29% (6)	39% (12)	0.6
Total number of IUTs in current pregnancy	5.3 ± 1.9	2.9 ± 2.2	**<0.001**
GA at last IUT	35.3 ± 0.8 (34.1–36.4)	30.6 ± 3.2 (20.0–33.6)	**<0.001**

Data are presented as % (n) or mean ± SD; Significance for differences was measured using the chi-square test, with Fisher’s exact test applied when expected frequencies were less than 5. GA, gestational age; IUT, intra uterine transfusion; MCA PSV, middle cerebral artery peek systolic velocity; MOM, multiple of the median. *Other indications for IUT include cases of anti-Kpb, anti-c, non-immune causes, pancytopenia, and SPTA1 mutation. *Values in bold are statistically significant.

### Safety outcomes

Non-inferiority analysis demonstrated the safety of late IUT, with a trend toward lower procedure-related complications compared to early IUT (0% vs. 20.0%, *p* = 0.069). The late IUT group showed no cases of chorioamnionitis, placental abruption, or PPROM, compared to rates of 3.2, 3.2, and 13%, respectively, in the early group (Table 3). One neonatal death occurred in the early group following emergency delivery at 24 + 3 weeks in a parvovirus-affected pregnancy, where umbilical cord bleeding during the second transfusion necessitated intracardiac blood administration, resulting in IUFD 2 days later. No deaths occurred in the late IUT group.

**Table 3:** Obstetrical outcomes in late vs. early intrauterine transfusion groups.

Study group	Last IUT≥34 weeks of gestation; *n* = 21	Last IUT<34 weeks of gestation; *n* = 31	*p* value
GA at delivery	37.2 ± 1.06 (34.4–39.0)	34.1 ± 3.6 (24.3–41.0)	**<0.001**
Vaginal delivery	67% (14)	48% (15)	0.14
Planned CS	14% (3)	6.5% (2)	
Emergency CS	19% (4)	45% (14)	
Induction of labor	48% (10)	45% (14)	>0.9
Abruption	0% (0)	3.2% (1)	>0.9
Chorioamnionitis	0% (0)	3.2% (1)	>0.9
PPROM	0% (0)	13% (4)	0.14
Administration of steroids	67% (14)	82% (23)	0.3
GA at time of steroids administration	26.8 ± 1.9 (24.2–30.4)	27.2 ± 2.8 (23.4–33.4)	0.6
Interval between steroids administration and delivery (days)	60 ± 25	35 ± 26	**0.006**
Procedure related complications	0% (0)	20% (6)	0.07
Perinatal loss after IUT	0% (0)	3.2% (1)	>0.9

Data are presented as % (n) or mean ± SD; Significance for differences was measured using the chi-square test, with Fisher’s exact test applied when expected frequencies were less than 5. GA, gestational age; CS, cesarean section; PPROM, premature rupture of membranes; procedure related complications include premature rupture of membranes, placental abruption, chorioamnionitis, or fetal demise within 1 week post-procedure. Additionally, complications such as non-reassuring fetal monitoring, bleeding from the puncture site, or cord hematoma occurring within 24 h were also considered related to the procedure. *Values in bold are statistically significant.

### Procedural characteristics

Analysis of all procedures showed comparable technical parameters between groups, including use of fetal paralysis, anesthesia requirements, estimated fetal weight, pre-transfusion MCA-PSV values, and transfusion volumes. Pre-transfusion hematocrit values were also similar between groups.

To assess whether patient selection factors influenced the decision to extend IUT beyond 34 weeks, we compared the final pre-34-week procedures between groups. Procedural characteristics were largely comparable, including fetal weight, MCA-PSV values, pre-transfusion hematocrit, and transfusion volumes. The few observed differences—earlier timing (30.5 ± 3.2 vs. 32.1 ± 1.1 weeks, *p* < 0.05) and lower post-transfusion hematocrit (41.0 ± 5.6 vs. 46.3 ± 7.2, *p* < 0.05) in the late IUT group—reflect practice evolution toward more conservative management rather than case-specific factors influencing individual decisions (Supplementary Table 3).

### Obstetrical and neonatal outcomes

**Figure 2:**
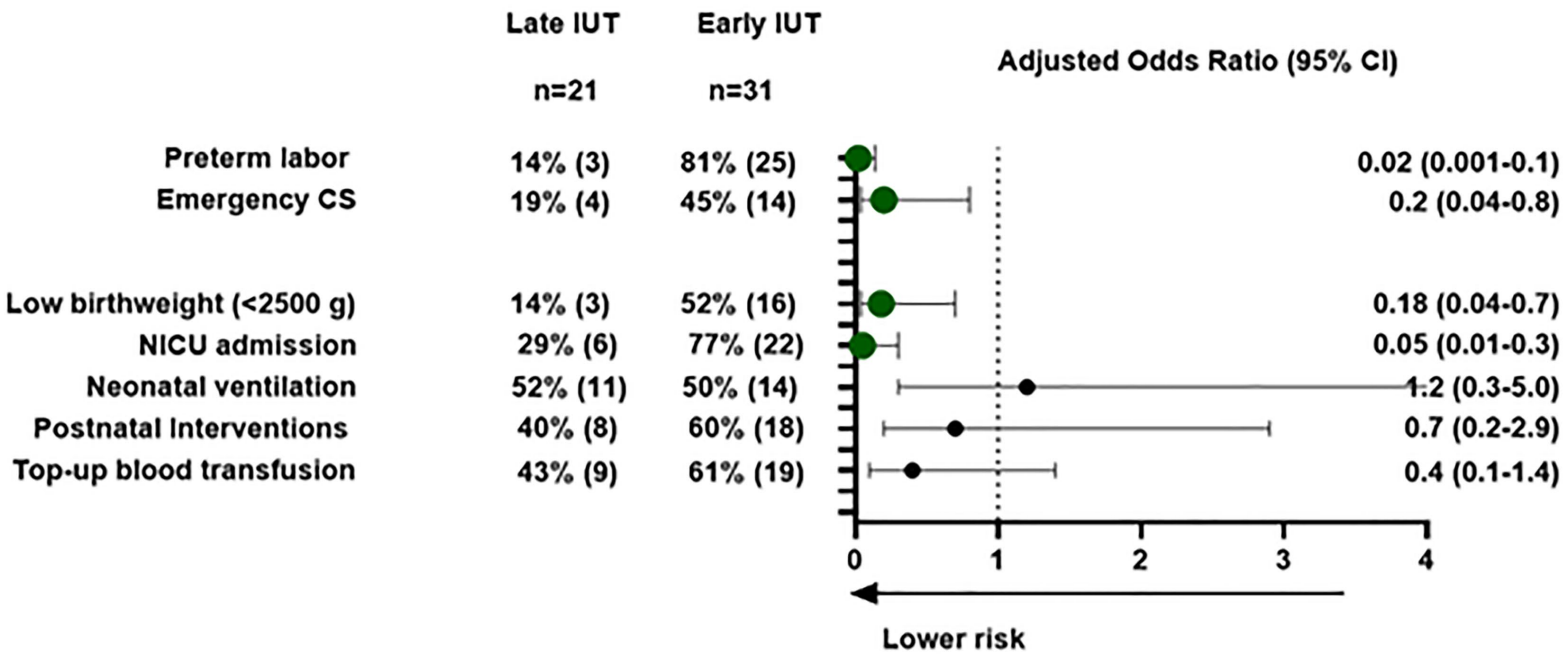
Maternal and neonatal outcomes for early vs. late intrauterine transfusions: a multivariable logistic regression analysis. Maternal and neonatal outcomes: multivariable logistic regression analysis. Forest plot showing adjusted odds ratios (OR) with 95% confidence intervals comparing late IUT (≥34 weeks) vs. early IUT (<34 weeks). Circles represent point estimates; horizontal lines show confidence intervals. Outcomes adjusted for maternal age, parity, previous IUT history, and gestational age at first IUT. Values <1.0 favor late IUT group Abbreviations: IUT, intra-uterine transfusion; NICU, neonatal intensive care unit. Postnatal interventions include phototherapy, intravenous immunoglobulin (IVIG), or top-up blood transfusion.

Late IUT was associated with significantly improved obstetrical outcomes, including higher mean gestational age at delivery (37.2 ± 1.06 vs. 34.1 ± 3.6 weeks, *p* < 0.001) and reduced emergency cesarean rates (19% vs. 45%; Table 3). In multivariate analysis, emergency cesarean section was specifically evaluated as an adverse outcome given its association with urgent clinical scenarios and potentially worse maternal-neonatal outcomes. After adjusting for maternal age, parity, previous IUT history, and gestational age at first IUT, late IUT maintained independent protective effects against preterm labor (adjusted OR 0.02, 95% CI: 0.001–0.14), emergency cesarean section (adjusted OR 0.2, 95% CI: 0.04–0.8), low birth weight (adjusted OR 0.18, 95% CI: 0.04–0.7), and NICU admission (adjusted OR 0.05, 95% CI: 0.01–0.3; Figure 2).

Primary analysis of all cases showed significantly better neonatal outcomes in the late IUT group, including higher birth weights (2,960 ± 399 g vs. 2,350 ± 620 g, *p* < 0.001), lower NICU admission rates (29% vs. 71%, *p* < 0.05), and higher mean hematocrit at delivery (Table 4).

**Table 4:** Neonatal outcomes in late vs. early intrauterine transfusion groups.

Study group	Last IUT≥34 weeks of gestation; *n* = 21	Last IUT<34 weeks of gestation; *n* = 31	*p* value
Birthweight	2,960 ± 399	2,350 ± 620	**<0.001**
Sex (male)	38% (8)	42% (13)	>0.9
APGAR >7 (first min)	95% (20)	77% (24)	0.12
Cord blood pH	7.34 ± 0.07	7.26 ± 0.1	**<0.05**
Neonatal hematocrit	44 ± 12	36 ± 13	**<0.05**
Neonatal bilirubin	9.0 ± 4.7	11.6 ± 5.8	0.12
NICU admission	29% (6)	71% (22)	**<0.05**
Neonatal ventilation	52% (11)	48% (14)	>0.9
Phototherapy	57% (12)	58% (18)	>0.9
Neonatal blood transfusion	43% (9)	61% (19)	0.3
IVIG	19% (4)	19% (6)	>0.9
Neonatal death	0% (0)	3.2% (1)	>0.9

Data are presented as % (n) or mean ± SD; Significance for differences was measured using the chi-square test, with Fisher’s exact test applied when expected frequencies were less than 5. *Values in bold are statistically significant.

The distribution of non-immune causes exclusively in the early IUT group reflects our classification system based on timing of the final IUT. Non-immune conditions typically require fewer transfusions, often completing treatment before 34 weeks regardless of delivery timing. Subgroup analysis of HDFN cases alone (Supplementary Tables 1, 2) confirmed that the benefits of late IUT persist in this homogeneous population, with additional benefits including significantly lower mean bilirubin levels (8.5 ± 4.4 vs. 12.1 ± 3.3, *p* < 0.01) and reduced need for neonatal top-up blood transfusion (40% vs. 85%, *p* < 0.05). This consistent pattern supports the benefits of late IUT particularly in alloimmunized populations.

## Discussion

### Principal findings

This study demonstrates both the safety and non-inferiority of extending IUT beyond traditional gestational age limits. Our hierarchical analysis established non-inferiority of late IUT regarding procedural complications following the last IUT prior to delivery, with a trend toward superiority in reducing procedure-related complications. We also demonstrated superiority in reducing prematurity-related outcomes, with higher birth weights in the late IUT group compared to the early IUT group.

### Results in the context of what is known

Current guidelines from major professional organizations vary in their recommendations for IUT timing. ACOG suggests performing the last IUT between 30 and 32 weeks with delivery at 32–34 weeks (9), while RCOG emphasizes individualized assessment and multidisciplinary planning, balancing the timing of delivery with the severity of fetal anemia and the potential need for fetal therapy (16). SOGC highlights the importance of delivering in tertiary care centers with comprehensive prenatal and postnatal planning (17). Our findings align with the recent trend toward delaying the last IUT and delivery, supporting the benefits of full-term delivery to optimize neonatal outcomes (18–20). This approach is consistent with the established “39-week rule,” which demonstrates optimal neonatal outcomes with delivery at or beyond 39 weeks in uncomplicated pregnancies (13, 14, 18, 19, 21).

Analysis of pre-34-week procedures revealed that the early IUT group had higher post-transfusion hematocrit levels, suggesting a more aggressive correction strategy in preparation for planned early delivery. In contrast, the late IUT group employed a more conservative approach that facilitated safer serial transfusions beyond 34 weeks. Importantly, the technical aspects of the procedures remained consistent between groups, indicating that outcome differences reflect management strategy rather than procedural variation.

Our findings demonstrate generalizability potential to high-volume fetal therapy centers with appropriate resources. As a national referral center, Hadassah’s high procedural volume, experienced operators, and integrated blood banking systems are comparable to major international centers. However, successful late IUT implementation requires experienced operators, comprehensive monitoring facilities, organized blood banking with rapid response capabilities, and robust neonatal care. Clinical guidelines may need resource-based stratification: high-resource centers can safely extend IUT beyond traditional limits, while resource-limited settings should concentrate cases in fewer specialized centers to maintain expertise and safety.

### Clinical implications

The timing of repeat transfusions presents a critical clinical dilemma: each additional IUT carries a 1.5–3% risk of fetal morbidity and mortality, yet early delivery poses significant prematurity-related risks. Our study demonstrates that extending IUT into the late preterm period can effectively balance these competing risks.

Management of late-gestation IUT requires specific adaptations to maintain safety. The reduced specificity of middle cerebral artery peak systolic velocity (MCA-PSV) measurements after 35 weeks necessitates a modified monitoring protocol, including more frequent assessments and integration of additional parameters. Our experience suggests that these adaptations effectively support decision-making in late gestation.

The evolution of our practice mirrors the broader shift in maternal-fetal medicine toward optimizing gestational age at delivery when safely possible. Following ACOG’s emphasis on the benefits of term delivery and guidelines addressing alloimmunization management, our center systematically implemented enhanced monitoring protocols and standardized patient selection criteria while maintaining consistent procedural technique.

### Research implications

While our current findings strongly support reconsidering traditional gestational age limits for IUT, future multi-center studies will help refine patient selection and monitoring protocols for late IUT. Additionally, long-term neurodevelopmental follow-up beyond 6 months is needed, although existing evidence on term delivery outcomes suggests that achieving later gestational ages through late IUT likely confers similar developmental and respiratory benefits.

Further research should also focus on optimizing the modified monitoring protocols required for late-gestation IUT, particularly given the reduced specificity of MCA-PSV measurements after 35 weeks. Standardized approaches to integrate additional monitoring parameters could improve decision-making in late gestation.

### Strengths and limitations

The study’s strengths include its comprehensive long-term follow-up, consistent procedural technique, detailed documentation of procedural parameters, systematic evaluation of both safety and efficacy outcomes, and robust statistical approach using hierarchical testing. Our integrated, comprehensive clinical follow-up ensures accurate documentation of all neonatal interventions and outcomes reported in our study. Additionally, our findings remained consistent in subgroup analyses of hemolytic disease of the fetus and newborn (HDFN) cases, supporting the generalizability of our conclusions.

Limitations include the retrospective design, potential selection bias, and inclusion of multiple pregnancies from the same women. While such repeated measures could theoretically affect our statistical analysis, the magnitude of improved outcomes across multiple parameters suggests the findings are robust despite this methodological constraint. The single-center nature and relatively small sample size, particularly in the late IUT group, warrant further validation. While practice patterns evolved during our study period (2005–2024), this reflects real-world implementation of evidence-based changes, though the concurrent evolution of NICU practices—particularly adoption of more restrictive neonatal transfusion thresholds—may have influenced some outcomes independent of our intervention. However, our findings remained consistent in subgroup analyses, and the primary procedural and delivery outcomes are less susceptible to these evolving practices.

## Conclusion

Our study demonstrates that extending IUT beyond 34 weeks is a safe and effective strategy for managing fetal anemia, offering significant benefits in reducing preterm birth-related complications while maintaining procedural safety. This evidence-based approach aligns with the broader trend in maternal-fetal medicine toward optimizing gestational age at delivery, suggesting that an individualized strategy extending IUT to 36 + 6 weeks can help achieve the well-documented benefits of term delivery in this high-risk population (14, 21).

## Data Availability

The raw data supporting the conclusions of this article will be made available by the authors, without undue reservation.
