# Supplementary figures and images for: Late vs. early intrauterine blood transfusion in fetal anemia: impact on maternal and neonatal outcomes
Source: Front Med (Lausanne). 2025 Sep 5;12:1614989. doi: 10.3389/fmed.2025.1614989 (PMC12446343; doi:10.3389/fmed.2025.1614989)

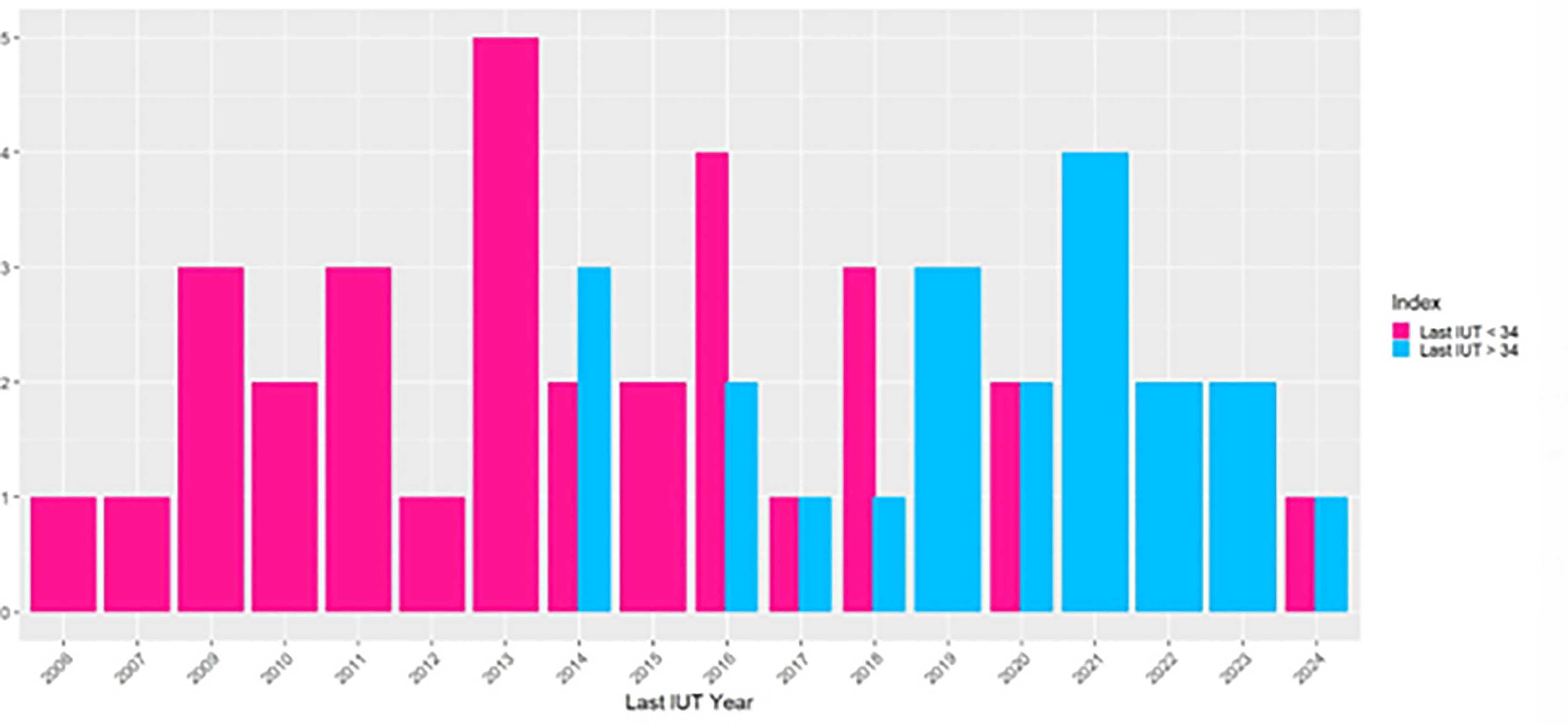

Supplement: Supplementary file 1 [file Image_1.TIF]
