# Supplementary material for: Late vs. early intrauterine blood transfusion in fetal anemia: impact on maternal and neonatal outcomes
Source: Front Med (Lausanne). 2025 Sep 5;12:1614989. doi: 10.3389/fmed.2025.1614989 (PMC12446343; doi:10.3389/fmed.2025.1614989)
Supplement: Supplementary file 2 [file Table_1.DOCX]

Supplementary Table 1: Obstetrical Outcomes in Late vs. Early Intrauterine Transfusion Groups: HDFN-Specific Analysis

|  | **Last IUT≥34 weeks of gestation**  **n=20** | **Last IUT<34 weeks of gestation**  **n=20** | ***p* value** |
| --- | --- | --- | --- |
| GA at delivery | 37.2 ± 1.03  (34.4-39.0) | 34.7 ± 2.1  (29.5-37.2) | **<0.001** |
| Vaginal delivery | 70% (14) | 45% (9) | 0.3 |
| Planned CS | 10% (2) | 10% (2) |  |
| Emergency CS | 20% (4) | 45% (9) |  |
| Induction of labor | 50% (10) | 45% (9) | >0.9 |
| Abruption | 0% (0) | 5.0% (1) | >0.9 |
| Chorioamnionitis | 0% (0) | 0% (0) | >0.9 |
| PPROM | 0% (0) | 10% (2) | 0.5 |
| Administration of steroids | 65% (13) | 89% (17) | 0.13 |
| GA at time of steroids administration | 26.8 ± 2.07  (24.2-30.4) | 27.3 ± 3.1  (23.4-33.4) | 0.6 |
| Interval between steroids administration and delivery (days) | 63 ± 24 | 41 ± 27 | 0.03 |
| Procedure related complications | 0% (0) | 21% (4) | 0.11 |
| Perinatal loss after IUT | 0% (0) | 0% (0) | >0.9 |

Data are presented as % (n) or mean ± SD; Significance for differences was measured using the chi-square test, with Fisher’s exact test applied when expected frequencies were less than 5. GA, gestational age; CS, cesarean section; PPROM, premature rupture of membranes; procedure related complications include premature rupture of membranes, placental abruption, chorioamnionitis, or fetal demise within one week post-procedure. Additionally, complications such as non-reassuring fetal monitoring, bleeding from the puncture site, or cord hematoma occurring within 24 hours were also considered related to the procedure.
