# Supplementary material for: Late vs. early intrauterine blood transfusion in fetal anemia: impact on maternal and neonatal outcomes
Source: Front Med (Lausanne). 2025 Sep 5;12:1614989. doi: 10.3389/fmed.2025.1614989 (PMC12446343; doi:10.3389/fmed.2025.1614989)
Supplement: Supplementary file 3 [file Table_2.DOCX]

**Supplementary Table 2: Neonatal Outcomes in Late vs. Early Intrauterine Transfusion Groups: HDFN-Specific Analysis**

|  | **Last IUT≥34 weeks of gestation**  **n=20** | **Last IUT<34 weeks of gestation**  **n=20** | ***p* value** |
| --- | --- | --- | --- |
| Birthweight | 2987 ± 388 | 2483 ± 392 | **<0.001** |
| Sex (male) | 35% (7) | 55% (11) | 0.2 |
| APGAR >7 (first min) | 95% (19) | 85% (17) | 0.12 |
| Cord blood Ph | 7.34 ± 0.07 | 7.26 ± 0.1 | **<0.05** |
| Neonatal hematocrit | 45 ±12 | 33 ± 11 | **<0.05** |
| Neonatal bilirubin | 8.5 ± 4.4 | 12.1 ± 3.3 | **<0.05** |
| NICU admission | 25% (5) | 80% (16) | **<0.001** |
| Neonatal ventilation | 50% (10) | 45% (9) | >0.9 |
| Phototherapy | 55% (11) | 65% (13) | 0.7 |
| Neonatal blood transfusion | 40% (8) | 75% (15) | **<0.05** |
| IVIG | 20% (4) | 30% (6) | 0.7 |
| Neonatal death | 0% (0) | 0% (0) | >0.9 |

Data are presented as % (n) or mean ± SD; Significance for differences was measured using the chi-square test, with Fisher’s exact test applied when expected frequencies were less than 5.
