# Supplementary material for: Late vs. early intrauterine blood transfusion in fetal anemia: impact on maternal and neonatal outcomes
Source: Front Med (Lausanne). 2025 Sep 5;12:1614989. doi: 10.3389/fmed.2025.1614989 (PMC12446343; doi:10.3389/fmed.2025.1614989)
Supplement: Supplementary file 4 [file Table_3.docx]

**Supplementary Table 3: Comparison of final Pre-34 Week Intrauterine Transfusion Characteristics Between Early and Late IUT Management Strategies**

|  | **Last IUT≥34 weeks of gestation**  **n=21** | **Last IUT<34 weeks of gestation**  **n=31** | ***p* value** |
| --- | --- | --- | --- |
| Gestational age at IUT | 32.1 ± 1.1 | 30.5 ± 3.2 | **<0.05** |
| Fetal paralysis | 21% (3) | 5.3% (1) | 0.3 |
| Local or regional anesthesia | 14% (3) | 4.8% (1) | 0.6 |
| Fetal weight | 1992 ± 328 | 1720 ± 680 | 0.14 |
| MCA PSV value before IUT (mom) | 1.4 ± 0.2 | 1.6 ± 0.3 | 0.09 |
| MCA PSV value after IUT (mom) | 0.8 ± 0.2 | 0.9 ± 0.2 | 0.7 |
| Hematocrit value before IUT | 25.2 ± 5.6 | 22.4 ± 6.3 | 0.12 |
| Hematocrit value after IUT | 46.3 ± 7.2 | 41.0 ± 5.6 | **<0.05** |
| Volume of blood transfused (ml) | 82 ± 16 | 72 ± 30 | 0.2 |

This table compares the characteristics of the last IUT performed before 34 weeks gestation in both groups.
Data are presented as % (n) or mean ± SD; Significance for differences was measured using the chi-square test, with Fisher’s exact test applied when expected frequencies were less than 5. IUT; intrauterine transfusion, MCA; middle cerebral artery, PSV; peak systolic velocity**.**
